# Using computed tomography enterography to evaluate patients with Crohn's disease: what impact does examiner experience have on the reproducibility of the method?

**DOI:** 10.1590/0100-3984.2015.0131

**Published:** 2017

**Authors:** Stênio Burlin, Larissa Rossini Favaro, Elisa Almeida Sathler Bretas, Lincoln Seiji Taniguchi, Ana Paula Loch, Marjorie Costa Argollo, Orlando Ambrogini Junior, Giuseppe D'Ippolito

**Affiliations:** 1 MD, Radiologist in the Department of Diagnostic Imaging of the Escola Paulista de Medicina da Universidade Federal de São Paulo (EPM-Unifesp), São Paulo, SP, Brazil.; 2 MD, Radiologist, Specialist in Abdominal Imaging in the Department of Diagnostic Imaging of the Escola Paulista de Medicina da Universidade Federal de São Paulo (EPM-Unifesp), São Paulo, SP, Brazil.; 3 MD, Radiologist, Graduate Student in Abdominal Imaging in the Department of Diagnostic Imaging of the Escola Paulista de Medicina da Universidade Federal de São Paulo (EPM-Unifesp), São Paulo, SP, Brazil.; 4 Pharmacist, Graduate Student in Preventive Medicine at the Faculdade de Medicina da Universidade de São Paulo (FMUSP), São Paulo, SP, Brazil.; 5 MD, Graduate Student in Clinical Gastroenterology, Discipline of Clinical Medicine, Department of Medicine of the Escola Paulista de Medicina da Universidade Federal de São Paulo (EPM-Unifesp), São Paulo, SP, Brazil.; 6 PhD, Affiliate Professor, Discipline of Clinical Medicine, Department of Medicine of the Escola Paulista de Medicina da Universidade Federal de São Paulo (EPM-Unifesp), São Paulo, SP, Brazil.; 7 Tenured Adjunct Professor in the Department of Diagnostic Imaging of the Escola Paulista de Medicina da Universidade Federal de São Paulo (EPM-Unifesp), São Paulo, SP, Brazil.

**Keywords:** Reproducibility of results, Crohn disease, Tomography, X-ray computed

## Abstract

**Objective:**

To assess the impact that examiner experience has on the reproducibility and accuracy of computed tomography (CT) enterography in the detection of radiological signs in patients with Crohn's disease.

**Materials and Methods:**

This was a retrospective, cross-sectional observational study involving the analysis of CT enterography scans of 20 patients with Crohn's disease. The exams were analyzed independently by two radiologists in their last year of residence (duo I) and by two abdominal imaging specialists (duo II). The interobserver agreement of each pair of examiners in identifying the main radiological signs was calculated with the kappa test. The accuracy of the examiners with less experience was quantified by using the consensus among three experienced examiners as a reference.

**Results:**

Duo I and duo II obtained a similar interobserver agreement, with a moderate to good correlation, for mural hyperenhancement, parietal thickening, mural stratification, fat densification, and comb sign (kappa: 0.45-0.64). The less experienced examiners showed an accuracy > 80% for all signs, except for lymph nodes and fistula, for which it ranged from 60% to 75%.

**Conclusion:**

Less experienced examiners have a tendency to present a level of interobserver agreement similar to that of experienced examiners in evaluating Crohn's disease through CT enterography, as well as showing satisfactory accuracy in identifying most radiological signs of the disease.

## Abstract (pt)

**Objetivo:**

Avaliar o impacto da experiência do examinador na reprodutibilidade e na acurácia de detecção de sinais radiológicos em exames de enterografia por tomografia computadorizada (entero-TC) de pacientes portadores da doença de Crohn.

**Materiais e Métodos:**

Análise retrospectiva de exames de entero-TC de 20 pacientes com doença de Crohn. Os exames foram analisados, independentemente, por dois radiologistas do último ano de residência (dupla I) e dois especialistas em imagem abdominal (dupla II). A concordância interobservador de cada dupla de examinadores para a identificação dos principais sinais tomográficos foi calculada pelo teste kappa. A acurácia da dupla I foi medida usando-se como referência um consenso entre três examinadores experientes.

**Resultados:**

Ambas as duplas obtiveram concordância interobservador semelhante e com correlação moderada a boa para os sinais de estratificação mural, espessamento da parede, realce mucoso, densificação da gordura e sinal do pente (kappa: 0,45-0,64). Os examinadores menos experientes apresentaram acurácia superior a 80% para os sinais, exceto para linfonodos e fístula (entre 60% e 75%).

**Conclusão:**

Examinadores menos experientes têm tendência a apresentar grau de concordância entre si semelhante à de examinadores experientes na avaliação dos principais sinais tomográficos da doença de Crohn, com acurácia considerada satisfatória, para a maioria dos sinais.

## INTRODUCTION

Crohn's disease is one of the most prevalent inflammatory bowel diseases. It is a noncaseating, granulomatous disease, of uncertain origin, that can lead to inflammation of any part of the digestive tract, the terminal ileum being the most commonly affected site. The disease occurs transmurally, with discontinuous focal impairment of diverse parts of the gastrointestinal tract, in some cases inducing the formation of stenosis with intestinal obstruction and fistulas, among other complications^(1)^. In the assessment of patients with Crohn's disease, the radiologist plays a crucial role, not only in assisting in the initial diagnosis, via the definition of the extent of the disease, but also by establishing the degree of inflammatory activity and its associated complications^(2,3)^.

Diverse imaging methods can be used in order to assess Crohn's disease, among which are magnetic resonance (MR) enterography and computed tomography (CT) enterography. Although MR enterography presents the main advantage of not using ionizing radiation, it has certain limitations, such as lower availability and lower reproducibility, in comparison with CT enterography, mainly the diagnosis of the complications of the disease and during its acute phase^(3-5)^. For these reasons, CT enterography has been recommended as a valuable diagnostic option in the initial evaluation of patients suspected of having Crohn's disease and in the definition of its complications^(2-5)^.

Numerous studies have shown good interobserver agreement in the evaluation of Crohn's disease with CT enterography ^(3-5)^. However, studies that establish the impact of examiner experience on the reproducibility of the method are still lacking. Only one study of MR enterography quantified the impact of examiner experience on the evaluation of the main signs of Crohn's disease and concluded that more experienced radiologists tend to have better performance in the evaluation of vascular engorgement (designated the comb sign) and of lymph node enlargement^(6)^. Therefore, we do not know exactly whether, among less experienced radiologists and specialists in abdominal imaging, CT enterography shows similar accuracy and reproducibility (good interobserver agreement).

The objective of this study is to assess the level of agreement between examiners with lesser and greater degrees of experience in the interpretation of the main symptoms of Crohn's on CT enterography, as well as determining the accuracy of these young professionals.

## MATERIALS AND METHODS

This was a cross-sectional, retrospective, observational study, undertaken at a single institution between January 2012 and December 2013. The study was approved by the Research Ethics Committee of the Federal University of São Paulo Paulista School of Medicine. All patients gave written informed consent.

We included 20 consecutive patients with a clinical diagnosis of Crohn's disease, all of whom were followed at the inflammatory bowel disease outpatient clinic and in whom CT enterography was indicated because of suspicion of inflammatory activity or complications related to the disease.

The exclusion criteria were as follows: patients who refused to participate in the study; having a history of severe allergic reaction to iodinated contrast medium; having renal failure; and presenting with any other contraindication to abdominal CT involving the use of iodinated contrast medium.

Of the 20 patients evaluated, 6 (30%) were female and 14 (70%) were male. Ages ranged from 28 to 65 years (mean, 39.5 years). The mean time since the onset of symptoms was 11.2 years (range, 1-17 years). On average, the disease had been diagnosed 8.3 years before the patient underwent CT enterography.

### CT enterography procedure

We carried out all the examinations with a 64-channel multislice CT scanner (Brilliance 64; Philips Medical Systems, Best, The Netherlands). The multislice technique and volumetric acquisition were used, extending from the diaphragm to the pubic symphysis, 50 s after the start of the intravenous injection of the contrast medium^(7)^. The contrast medium was administered with an automated injection pump, at a speed of 3 mL/s, in a volume of 2 mL/kg of body weight, with a maximum total volume of 150 mL. The following technical parameters were used in the CT examinations: collimation, 64 × 0.624; pitch, 0.891; slice thickness, 1 mm and 3 mm; tube voltage, 120 kVp; and tube current, variable (depending on the abdominal thickness of the patient). To determine the radiation dosage, we used dose modulation software^(8)^. In terms of the endoluminal contrast, the CT enterography protocol involved oral administration of a polyethylene glycol solution, as previously reported^(9,10)^. The examinations and image acquisition took place 45 min after the initiation of the oral administration of the contrast medium^(11)^.

### Examiners

Four examiners, working independently and blinded to the patient clinical data, interpreted the CT enterography examinations, using a picture archiving and communication system (Synapse; Fujifilm Medical Systems, Stamford, CT, USA), on the workstation provided with the CT equipment. Of the four examiners, two were third-year residents in general radiology and diagnostic imaging (duo I) and two were radiologists specializing in abdominal imaging with more than five years of experience in performing CT enterography (duo II). The examiners with less experience had previously received a short training course, evaluating the CT enterography examinations of ten Crohn's disease patients who were not included in this study. A third specialist in abdominal imaging, with 25 years of experience, subsequently evaluated in consensus with the experienced examiners the CT enterography examinations, determining the presence or absence of the main radiological signs related to Crohn's disease. This consensual evaluation was used as the reference for the calculation of the accuracy of duo I.

### Radiological signs

**Figure 1:**
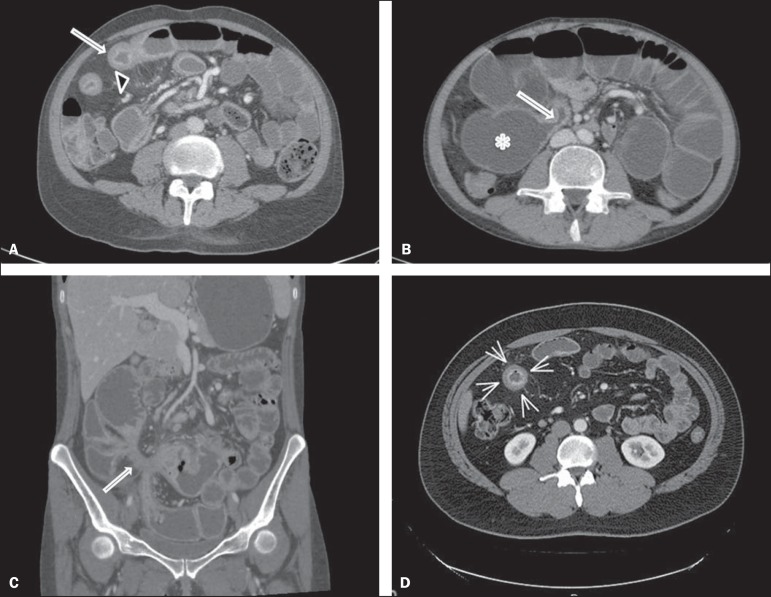
**A:** Note the thickened small loop with mucous enhancement (arrow) and the comb sign (arrowhead), corresponding to engorgement of the mesenteric arcade. **B:** The arrow indicates the area of ileal stenosis, with upstream dilation of the loops (asterisk). **C:** Note the fistula (arrow), characterized by a starred image corresponding to its pathway, creating a communication through the bowel loops in the right iliac fossa. **D:** In the region indicated by the arrows, note the mural stratification in the ileal loop, together with mucous enhancement, allowing us to distinguish among the various parietal layers.

We evaluated the presence of the main radiological signs of Crohn's disease, its inflammatory activity and its complications (Figure 1), defined as the following^(12-15)^:

- Wall thickening: thickening of the intestinal wall ≥ 5 mm.
- Mucous enhancement: visual comparison with healthy adjacent loops.
- "Comb sign": corresponding to the *vasa recta* being dilated from the inflammatory process.
- Densification of the fat adjacent to the loop.
- Regional lymph node enlargement: lymph nodes measuring ≥ 5 mm along their short axis. We also measured the number of enlarged lymph nodes identified.
- Presence of stenosis: reduction of the caliber of the loop with upstream distension, characterized by the small loop segment having a cross-sectional diameter > 2.5 cm, with or without collapse of the distal fragment.
- Presence of fistula: defined as a linear image, with peripheral enhancement, connecting two anatomical structures, with or without air or fluid content.

### Systematization of the analysis of images

The radiological signs described above were evaluated in a segmented and independent manner in the following intestinal segments: jejunum, ileum, right colon, transverse colon, and left colon. Therefore, for each patient, the presence or absence of the radiological signs were evaluated five times, or rather, in five segments. Consequently, a total of 100 intestinal segments were evaluated.

Due to the difficulty of analyzing the interpretation of lymph node enlargement and intestinal fistulas in a segmented manner, we interpreted those variables only as present or absent in each participant and not per intestinal segment.

### Calculation of reproducibility

We calculated interobserver agreement for the set of examiners as a group, as well as between the two inexperienced examiners (duo I) and the two experienced examiners (duo II), using the kappa (κ) test and grading agreement as follows: κ: 0.00-0.20, poor; κ: 0.21-0.40, weak; κ: 0.41-0.60, moderate; κ: 0.61-0.80, good; κ: 0.81-1.00, excellent.

### Calculation of accuracy

The sensitivity, specificity, and accuracy of the examiners in duo I, in terms of the detection of radiological signs of Crohn's disease on CT enterography, were calculated using the consensus analysis carried out by the three experienced examiners as a reference.

## RESULTS

### Prevalence of the findings

According to the overall evaluation of the examiners, the following symptoms were encountered: densification of the wall, in 22% of the intestinal segments evaluated; thickening of the wall, in 24%; prominent mucous increase, in 28%; mural stratification, in 27%; the comb sign, in 26%; and stenosis, in 20%. All these symptoms were most prevalent in the ileum. The prevalence of fistula and lymph node enlargement occurred, respectively, in 65 and 50% of patients studied.

### Measure of reproducibility

The two pairs of examiners presented similar results for reproducibility. There was moderate to good interobserver agreement (a strong correlation) between the two less experienced examiners (duo I), as well as between the two more experienced examiners (duo II), in the assessment of the majority of radiological signs of Crohn's disease, with the exception of lymph node enlargement and fistulas, for which there was poor interobserver agreement (a weak correlation) in both analyses (Table 1).

**Table 1:** Kappa test evaluation of agreement between examiners, by level of experience.

	Inexperienced			Experienced			Total
Variable	κ	95% CI		κ	95% CI		κ
Densification	0.62	0.44–0.80		0.60	0.42–0.79		0.61
Wall thickness	0.45	0.33–0.57		0.38	0.28–0.48		0.41
Mucous enhancement	0.55	0.37–0.73		0.69	0.51–0.87		0.62
Stratification	0.64	0.46–0.82		0.60	0.42–0.79		0.57
Comb sign	0.55	0.37–0.73		0.58	0.38–0.77		0.57
Stenosis	0.56	0.36–0.74		0.63	0.44–0.82		0.54
Fistula	0.44	0.07–0.81		0.19	0–0.45		0.33
Lymph node number	0.31	0–0.68		0.06	0–0.48		0
Lymph node size	0.09	0–0.46		0.17	0–0.60		0

95% CI, 95% confidence interval.

### Measure of accuracy

The less experienced examiners (duo 1) presented sensitivity, specificity, and accuracy above 80% for the variables mural densification, the comb sign, wall thickening, and mucous enhancement. Accuracy in the analysis of lymph nodes and fistulas ranged from 60% to 75% (Table 2).

**Table 2:** Sensitivity, specificity and accuracy of the less experienced examiners (examiners A and B), the consensus evaluation of the experienced examiners being used as a reference.

	Sensitivity			Specificity			Interobserver agreement	
Characteristic	%	95% CI		%	95% CI		κ	95% CI
Densification							0.62	0.44–0.80
A	90.5	71.1–97.4		89.9	81.3–94.8			
B	71.4	50.0–86.2		92.4	84.4–96.5			
Wall thickness							0.45	0.33–0.57
A	73.1	53.9–86.3		93.2	85.1–97.1			
B	76.9	58.0–89.0		91.0	81.7–95.3			
Mucous enhancement							0.55	0.37–0.73
A	89.3	72.8–96.3		87.0	77.7–92.8			
B	71.4	52.9–84.8		85.7	76.2–91.8			
Stratification							0.64	0.46–0.82
A	48.2	30.1–66.0		98.6	92.6–99.8			
B	66.7	47.8–81.4		97.3	90.1–99.3			
Comb sign							0.55	0.37–0.73
A	88.0	70.0–95.8		96.0	88.9–98.6			
B	56.0	37.1–73.3		94.7	87.1–97.9			
Stenosis							0.56	0.36–0.74
A	45.0	25.8–65.8		96.3	89.6–98.7			
B	50.0	29.9–70.1		98.8	93.3–99.8			
Fistula							0.44	0.07–0.81
A	42.9	21.4–67.4		100	61.0–100			
B	64.3	38.8–83.7		50.0	18.8–81.2			
Lymph node number							0.31	0.00–0.68
A	91.0	62.3–98.4		22.2	6.32–54.7			
B	100	74.1–100		22.2	6.32–54.7			
Lymph node size							0.09	0.00–0.46
A	63.6	35.4–84.8		44.4	18.8–73.3			
B	100	74.1–100		44.4	18.8–73.3			

95% CI, 95% confidence interval.

## DISCUSSION

The main objective of this study was to evaluate the reproducibility of CT enterography, which is simply the extent to which examiners agree between or among themselves. The evaluation of interobserver agreement is important to the validation of the use of the technique, because we expect that similar results can be reproduced in a consistent manner for different examiners. It is also crucial to establish what impact the experience of these examiners has on the reproducibility of the method, which can increase the robustness of its use in the diagnosis and monitoring of a given disease^(16,17)^. In the present study, we found that examiner inexperience had little influence on the reproducibility of CT enterography in the identification of the main radiological signs of Crohn's disease, as long as a short training course covering the method and disease was undertaken. The accuracy obtained by these examiners was considered satisfactory for all of the radiological signs, except for lymph node enlargement and fistula.

There have been few studies in which the main objective was to measure the reproducibility of CT enterography in the evaluation of patients with Crohn's disease^(4,18)^. This evaluation is made, generally, in a secondary manner, which can cause the results to diverge across studies. For example, some studies have reported excellent agreement for the detection of signs of Crohn's disease^(3,19)^, whereas others have reported only moderate agreement for the majority of symptoms^(4,18)^, with results similar to those obtained in the present study. This discrepancy can be explained by a number of variables, among which we can cite differences in examination protocols, in the quality of images obtained, and in the severity of the disease among the patients included in the study sample, heightened by the fact that radiological signs utilized have a certain degree of subjectivity, which requires their validation in reproducibility studies.

In the present study, for the variables that indicate the inflammatory activity of Crohn's disease, such as wall thickening, mucous enhancement, stratification, densification of the mesenteric adipose tissue, the comb sign, and stenosis, the agreement between the two inexperienced examiners was similar to that observed between the two more experienced examiners. This is in agreement with the results obtained by other authors who also quantified the reproducibility of CT enterography among specialists in abdominal imaging^(18)^. Our results suggest that the experience of the examiner has little impact on the reproducibility of CT enterography in the evaluation of patients with Crohn's disease. Our results are also similar to those obtained by other researchers in regard to the comb sign, wall stratification, and mucous enhancement criteria, as evaluated by experienced examiners^(4)^.

As described in a similar study quantifying the reproducibility of CT enterography in evaluating Crohn's disease^(6)^, we observed poor agreement in the evaluation of mesenteric lymph nodes, which could reflect the difficulty and subjectivity of evaluating this variable with CT enterography and MR enterography. However, unlike MR enterography, CT enterography showed good agreement in the evaluation of diverse signs of inflammatory activity of the disease, indicating a tendency toward improvement in the reproducibility of the method.

In the present study, the level of agreement between the experienced examiners was surprisingly low in the evaluation of fistulas. The reported prevalence of fistula in patients with Crohn's disease is generally 10-30%^(20,21)^, considerably lower than the 60% observed in our patient sample. It is possible that the elevated prevalence of fistula in our sample led some examiners to overestimate or underestimate the symptom, resulting in the discrepancy of results between examiners and consequently in poor agreement. Detection of a fistula between the bowel loops by CT enterography uses subjective criteria and is difficult to substantiate, because surgical exploration is typically contraindicated in this patient population^(20,22)^, as well as because other, potentially more effective, diagnostic methods, such as barium follow-through, capsule endoscopy, and CT with oral administration of water-soluble iodized (positive) contrast medium, are not routinely employed^(20,23,24)^. That could explain our finding of poor agreement even between examiners with more experience, motivating us to search for signs that are more consistent with a diagnosis of internal fistula in Crohn's disease and to use orally administered contrast media in the CT studies conducted because of suspicion of internal fistula. It should be borne in mind that, despite their desirability, there have been few studies demonstrating the reproducibility and accuracy of barium follow-through or the reproducibility of CT with positive contrast media in the diagnosis of enteral fistula. However, some of the studies aimed at establishing the value of CT enterography in the evaluation of fistula have done so without determining the accuracy of the method or its reproducibility or by studying samples of patients with known fistula, which reduces the strength of the CT enterography results^(20,24)^. Further studies, utilizing reliable reference standards, will be needed in order to evaluate not only the reproducibility but also the accuracy of CT enterography in the diagnosis of enteroenteric fistulas.

This study presents certain limitations. First, the patient sample was small. However, all of the patients evaluated presented an advanced stage of the disease with multiple radiological signs, increasing the prevalence of the variables analyzed. In addition, we used a reference standard based solely on a consensus between experienced examiners, although there is no surgical or histological gold standard, because surgical intervention is contraindicated in the majority of patients with Crohn's disease^(24-26)^. Furthermore, the study was carried out in a retrospective manner. Nevertheless, the homogeneity in the form of CT enterography acquisition, which has been included in the protocols of previous studies, permitted a consistent analysis in examinations considered to be of good diagnostic quality. Moreover, because of a lack of data corresponding to the period during which the CT enterography examinations were performed, we were unable to correlate the CT findings with clinical and laboratory changes in the patients (and therefore with the index of inflammatory activity). Further studies are warranted in order to determine the impact of examiner experience on the evaluation of the degree of inflammatory activity in Crohn's disease.

## CONCLUSION

In the present study, examiner experience was found to have no substantial impact on the reproducibility of CT enterography in the assessment of patients with Crohn's disease and a short training course was found to be adequate to obtain satisfactory accuracy in the detection of the main CT signs of the inflammatory activity of the disease. The diagnosis of enteral fistula by CT enterography remains a challenge that merits improvement in acquisition techniques, better examination protocols, and more examiner training.
